# Determinants of choosing in-kind benefits over cash: A study among Nepalese farmers

**DOI:** 10.1371/journal.pone.0300129

**Published:** 2024-07-11

**Authors:** Binod Khanal, Shuresh Ghimire, Pramila Wagle

**Affiliations:** 1 Prairie View A&M University, Prairie View, TX, United States of America; 2 University of Connecticut, Vernon, CT, United States of America; 3 Government of Nepal Ministry of Agriculture and Livestock Development, Department of Agriculture, Harihar Bhawan, Lalitpur, Nepal; University of Agriculture Faisalabad, PAKISTAN

## Abstract

This study investigates the determinants of choosing in-kind benefits over cash transfers when their respective values are equivalent. Employing a rigorous two-step experiment with a large sample size (n = 962), we offer real monetary rewards to respondents. In the first step, we asked whether the respondents would choose NRs. 1,000 (≈ US dollars 9) in cash or in-kind benefit that is worth NRs. 1,000. We observe that approximately two-thirds of participants opt for in-kind benefits of equal value to the proposed cash transfer. In analyzing the factors influencing this preference, our results indicate that households with higher non-farm incomes are less likely to choose in-kind benefits. Increasing the non-farm income by NRs. 100,000 respondents are 0.2% less likely to choose in-kind benefits. Furthermore, households with limited savings demonstrate a higher preference toward in-kind benefits over cash transfers. Not having NRs. 25,000 savings would make respondents 10% more likely to choose in-kind benefits. Previously receiving in-kind benefits also increase the likelihood of choosing them over cash. Additionally, households with restricted market access are more inclined to opt for in-kind benefits. Notably, in the second step of the experiment which involves only those who chose cash in the first step of the experiment, only 48% of respondents would opt for in-kind benefits even when values were higher by NRs. 150 to 450. This research sheds light on the factors affecting the decision-making process between in-kind benefits and cash transfers and provides insights into the design of effective social welfare policies. More specifically, findings from this study suggest tailored approaches for assisting people could be followed based on their income level and accessibility to the market.

## 1. Introduction

The implementation of anti-poverty programs commonly involves providing households or individuals with cash or in-kind benefits [1]. These transfers have been recognized for their significant welfare impacts [2]. In-kind benefits have shown greater effectiveness among households in need [3–6], while allowing less needy households to opt out of the program [7, 8].

Conversely, cash transfers afford recipient households the flexibility to optimize their utility by accessing a wide range of goods and services, such as education [9], health, and nutrition [10]. Schwab [11] finds improved food security resulting from randomly assigned in-kind benefits and cash transfers, with dietary quality improving among those who received cash. However, supporting agencies have raised concerns regarding the potential use of cash for risky expenditures, such as alcohol, tobacco, and gambling. In-kind transfers, on the other hand, can be costly and susceptible to corruption and bureaucratic obstacles, particularly for impoverished farmers. National and international aid programs administering in-kind benefits have faced criticism for their paternalistic approach and distrust toward economically disadvantaged groups [12]. Although studies on the preference for the in-kind benefit over cash or vice-versa have a greater implication regarding their role in policy formulation, this topic has not received limelight. Thus, this study explores the socio-economic factors that affect the choice of one benefit types over the other. Further, this study also investigates the substitutability of cash and in-kind transfers. Doing so, this study attempts to make some policy recommendation for the benefit transfers in the context of developing economies like that in Nepal, India, or several Asian countries.

Theoretical economic models predict a weak preference for cash over in-kind benefits, as cash offers greater expenditure options compared to the restricted choices imposed by in-kind transfers [13]. The findings from empirical studies show the varying preference and effectiveness of cash and in-kind transfers. Using experimental data from Mexico, Skoufias et al. [3] demonstrate that a cash transfer amounting to less than 25% of the cash value of an equivalent in-kind benefit could yield an equivalent impact on poverty reduction. Related to our study, Ghatak et al. [14] study preferences for in-kind benefits versus cash transfers in Bihar, one of the poorest states in India, finding that 45% of participants preferred in-kind benefits while the remainder favored cash. Moreover, Ghatak et al. [14] show that the preference for cash was influenced by household liquidity constraints, as evidenced by lower-cost housing and larger family sizes.

Thus, this study contributes in the development economics and economic policy literature by addressing an important, yet unexplored, topic on the factors affecting the preference of in-kind benefits over cash benefits among farming households in Central and Western Nepal, a developing country. Closely related to this study is the study by Ghatak et al. [14] where the respondents were asked to choose between bicycle vs cash. This current study addresses concerns of farming households as this subsidies and benefit distribution in Nepal is mostly targeted for farmers, thus, this study is believed to contribute in this front. Using a hypothetical scenario, we also examine the substitutability of cash and in-kind transfers. The findings of this study provide valuable insights for academics and development organizations, shedding light on the factors that determine preferences for in-kind and cash benefits. More specifically, findings from this study will provide tailored approaches for assisting people in the context of agrarian economy as policymakers benefit from the evidence-based knowledge generated by this study.

## 2. Background

As a developing country, Nepal relies heavily on agriculture, a major source of income for 68% of its population and contributes 34% to the GDP [15]. However, the agricultural sector in Nepal is predominantly subsistence-based, characterized by high labor intensity and limited access to resources. Consequently, agricultural productivity remains low, and the prevalence of chronic malnutrition, or stunting, affects 36% of children [15]. In an effort to enhance agricultural production and productivity, the government has implemented various measures, albeit with mixed results. The Ministry of Agricultural Development has invested in extension facilities and provided subsidies and benefit transfers to farmers. For instance, fertilizer subsidies are substantial in Nepal, although irregular supply has emerged as a significant challenge [16]. Assisting impoverished households with rice and implementing other nutritional programs have been a long-standing practice in several parts of the country, with an increasing trend in recent years. Additionally, local authorities have started providing cash benefits to farmers as encouragement and support. However, the authors are not aware of any studies that could offer guidance to policymakers regarding the selection of either cash or in-kind benefits for assisting farmers. Consequently, this study aims to provide valuable evidence on recipient households’ preferences in order to inform policy decisions.

## 3. Economic framework

**Fig 1:**
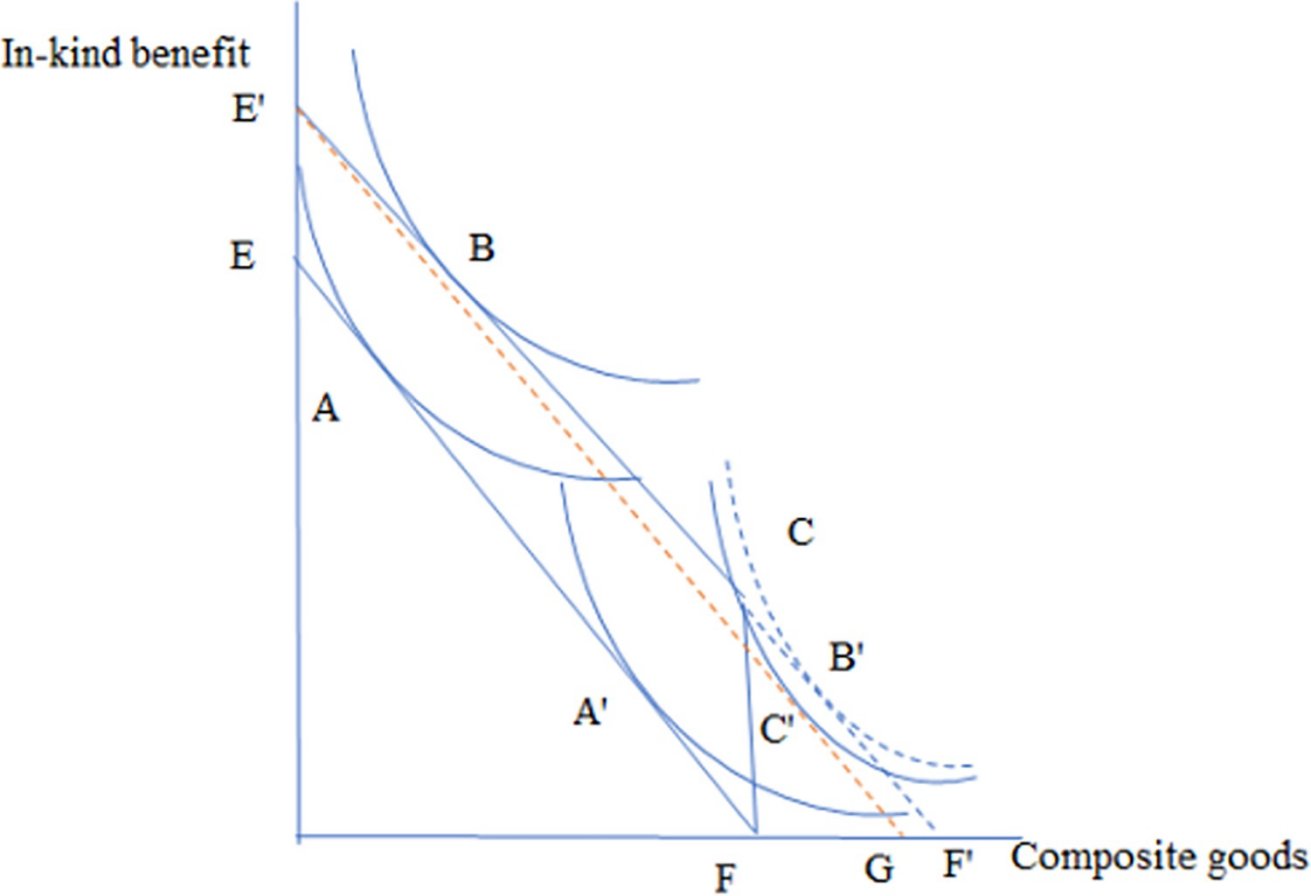
Utility of individuals under in-kind and cash benefit. EF is the original budget line for a household. With cash benefits, the new budget line is E`F`, and with in-kind benefits, the new budget line is E`CF. This figure is adapted from Currie and Gahvari (2008). For detailed theoretical concept on this, consult the original paper.

The economic framework of this model is distilled in Fig 1. EF is the original budget line for a household, showing how that household would spend its income. With cash benefits, the new budget line is E`F`, and with in-kind benefits, the new budget line is E`CF. There are two types of individuals: the first type—indifferent between cash and in-kind benefits like moving from A and B while increasing utility from the benefits. But the other type of individuals would be better off with cash benefits (A’ to B’) than with in-kind benefits who are constrained to remain at point C with lower level of utility (solid line with C’ tangent to the hypothetical budget line E’G). Empirically, different individuals have different levels of preference for cash transfers over in-kind benefits. This insinuates that some individuals might be willing to give up some in-kind transfer to get the less-than-equivalent cash benefit.

In this study, we empirically test what determine the choice of in-kind transfer over cash if the value of these transfers is same.

An individual’s choice of in-kind benefit over cash may depend on various factors like liquidity constraints, household income, and the usefulness of that in-kind in their household. For example, a liquidity constraint household might show a higher degree of preference for cash benefits over the in-kind benefits as they can utilize the cash to purchase the product of their need. Likewise, high income household could have already managed to have the necessary goods, this could prompt them to choose cash instead of in-kind benefit.

## 4. Research design and econometric model

We conducted an experiment among almost 962 farming households in 5 locations (Chitwan, Rupandehi, Palpa, Kapilvastu, and Kathmandu Valley) in Central and Western Nepal. We selected farmers from these regions purposively because they are leading suppliers of agricultural produce to major metro areas of the country—Kathmandu, Narayanghat, and Butwal.

The survey was conducted from January 25 to February 24, 2023 after it was exempted by the University of Connecticut Office of the Vice President for Research, Research Compliance Services/Institutional Review Board for the Human Subject Research (Protocol X22-0295). A written consent from participants was obtained before administrating the survey. The survey was designed based on five Key Informant Interview (KII). Before rolling out the final survey, it was pretested among the potential enumerators via Zoom. We surveyed 962 individuals for our survey where 954 respondents had full information for our analysis. The survey sample was determined so that it would ensure the desired power (approximately 80%) to the study. Our survey collected information on demographics, food security status, liquidity constraints, and experience with and substitutability of cash vs in-kind benefits. To determine the substitutability, we conducted a two-step experiment. In the first step, we asked, “Would you prefer NRs. 1000 (≈ US dollars 9) in cash or in-kind benefits worth NRs. 1000?” For those, who responded “Yes” to cash, we took them to the second step and asked the following follow-up question. “Suppose, we are giving you NRs. 800. However, you will have to invest that into one of the two ventures: the first one will give you NRs. 1000 in cash, while from the second venture, you will earn either fertilizer (or rice) worth NRs. 1300 (this value varies by treatment). Which venture will you choose?” The first step of this two-step experiment sorts out the respondents into two groups: (i) who prefer cash, and (ii) who prefer in-kind benefit if these benefits are of equal value. Further, only those who chose cash were transferred to the second experiment to see how much additional value would be needed for the respondents to switch to in-kind benefits. Thus, this process minimizes the biases in the substitutability of in-kind benefit vs cash benefit.

While the reward for the first step of the experiment was real, the second step was hypothetical. We initially proposed to reward the respondents by the value they choose in both steps of the experiment. However, the local leaders (the key informants) suggested that it could create conflict hampering the entire survey process since some respondents would go home with more money than others. Thus, to avoid the potential conflict, we rewarded NRs. 1,000 to each participant at the end of the experiment. However, the enumerators were trained to keep the experimental details secret until they reach the experiment section. The experiment in each location was conducted in a cluster of 20–25 individuals in a short time frame. These respondents were contacted by the help of farmers’ group through the contact of regional agricultural offices in each district. All cautionary actions were adopted to reduce the spillover of the information to other potential respondents.

The details of treatments with the cash and in-kind benefits are presented in Table 1. There are three levels of values of the in-kind benefit. Furthermore, we offered two types of in-kind products—rice and fertilizer. The respondents were randomly asked to choose between one of the products (rice and fertilizer) and one of the levels of valuations among NRs. 1150, NRs. 1300, and NRs. 1450. Since we used face-to-face interview as a survey method the randomization of the treatments was achieved in two steps. In the first step, we randomly stacked 6 different sets of printed questionnaires [2 (rice and fertilizer) x 3 (levels valuation of in-kind benefit) = 6]. In the second step, our enumerators randomly chose questionnaires from that stack to survey the respondents.

**Table 1:** Benefit types and treatments used in the step 2 of the experiment conducted on the determinants of choosing in-kind benefits over cash among Nepalese farmers in 2023.

Treatment	Cash	Fertilizer	Rice
	(1)	(2)	(3)
1	NRs. 1,000	NRs. 1,150	NRs. 1,150
2	NRs. 1,000	NRs. 1,300	NRs. 1,300
3	NRs. 1,000	NRs. 1,450	NRs. 1,450

The reason of making the base value NRs. 1000 is that the average daily wage for non-technical agricultural laborer is NRs. 1000 in Nepal. Likewise, in-kind benefits are fixed at 3 different values between NRs. 1000 and NRs. 1450 each with NRs. 150 difference. This difference is simply an hourly wage rate in Nepal rounded up to nearest NRs. 50. Further, we offered rice and fertilizer as the types of in-kind benefits to choose from because of their prime importance in Nepalese farming households. Nepalese agriculture sector has been facing deficiency of fertilizers in the supply chain (Ward et al., 2020). Many subsistence farmers are unable to use enough fertilizer because of credit/liquidity constraints. Due to the ongoing Ukraine-Russia conflict and other unknown factors, fertilizer supply was further affected and there was a shortage of fertilizers in Nepalese markets. Likewise, low income farmers are also among those who are food insecure [17]. After some months of harvest some farmers become food insecure, thus, could prefer rice of value more than NRs. 1000 over NRs. 1000 in cash.

We use an econometric model to find the factors affecting the preference of cash (instead of in-kind benefit of equal valuation) based on the first step of the experiment. The baseline model is given as:

$${Choose\_inkind}_{i}=\alpha_{0}+\alpha_{1}{CC}_{i}+\alpha_{2}{Dem}_{i}+\alpha_{3}{Off\_farm}_{i}+\alpha_{4}{FS}_{i}+\alpha_{5}{Market\_access}_{i}+\theta +\varepsilon_{i},$$ (1)

where *Choose*_*inkind*_*i*_ indicates a dummy variable where *Choose*_*inkind*_*i*_ = 1 if the individual chooses kind benefit instead of cash, otherwise 0. *CC* indicates variables related to credit constraint, *Off*_*farm* indicates off farm income, *Dem* indicates demographics, *FS* indicates food security status, and *Market_access* indicates the distance to nearest market. Demographic variables include gender (female = 1, 0 otherwise), caste (higher caste = 1, 0 otherwise), and schooling (high school or more = 1, 0 otherwise). The caste system in Nepal is a longstanding social structure that categorizes the population into various castes. Brahmin and Chhetri typically occupy the upper tiers of this hierarchy, while Janajati and Dalits are positioned differently within it. Food insecurity is defined as whether they experienced lack of food in the last month or whether they worry of experiencing lack of enough food in the last month. Those who fell into neither of these categories are considered food secure. We added location fixed effects *θ* (5 survey locations) to control for common attributes like geography, climate, and accessibility. *ε* is idiosyncratic error term.

We applied a linear probability model to Eq (1) to simplify the interpretation.

Based on the second step of the experiment, we analyze the substitutability between cash and in-kind transfers for the respondents using descriptive approach.

## 5. Data and results

### a. Descriptive statistics and expected signs

The descriptive statistics of the sample are shown in Table 2. The results show that 67% of the respondents prefer in-kind benefit worth of NRs. 1000 instead of NRs. 1000 in cash. This result is surprising, and much of our discussion will revolve around why this is the case. Our sample is less representative of females (42%) while it is over representative of *higher* caste—mostly Brahmin and Chhetri (62%). Since men in the Nepalese household control most of the income activities, we expect that women are more likely to prefer cash. Empirically, the relationship between the *higher* caste and choosing of in-kind can be of any sign. More than one-third of the respondents have completed secondary school level. Educated individuals can make more informed decisions. So, we expect the educated household to prefer cash instead of in-kind transfers. Because of the lack of proper record keeping among Nepalese farmers, the reported farm income may not be accurate. Therefore, we report only off-farm income. The average annual off-farm income is NRs. 272,000. We expect that high-earning households would prefer cash instead of in-kind benefit. Two-thirds of the respondents reported that they do not have NRs. 25 thousand in savings. Likewise, 20% reported that they consume low-quality goods and services because of credit constraints. We expect liquidity or credit-constrained respondents would prefer cash instead of in-kind benefit.

**Table 2:** The descriptive statistics of the samples in the study conducted on the determinants of choosing in-kind benefits over cash among Nepalese farmers in 2023.

Variables	Observations	Mean	SD
Choose in kind (1/0)	962	0.67	0.47
Female (1/0)	962	0.41	0.49
*Higher* caste (1/0)	962	0.62	0.49
Secondary school or more (1/0)	962	0.35	0.48
Off-farm income (in NRs. 100,000)	955	2.73	10.95
Lack NRs. 25, 000 in saving (1/0)	962	0.67	0.47
Low quality goods and services (1/0)	962	0.20	0.39
Food insecure (1/0)	962	0.28	0.45
Market distance (in 10 minutes)	962	2.27	2.05
Get cash (1/0)	962	0.19	0.39
Get in-kind (1/0)	961	0.58	0.49
**Regions (%)**			
Chitwan	962	22	
Kathmandu	962	14	
Kapilvastu	962	10	
Palpa	962	40	
Rupandehi	962	14	

Twenty eight percent of the households reported facing some kind of food insecurity. Empirically, the relationship between the food insecure and choosing in-kind can be of any sign. On average, the respondents have to walk almost 23 minutes to the nearest market. We expect that living farther from the market will make people choose in-kind benefit. This will reduce the associated transaction cost. Nineteen percent of the respondents reported receiving some form of cash benefits previously from other organizations. In comparison, 58% reported receiving some form of in-kind benefit. We expect that those who have received cash transfers in the past would likely prefer cash transfers, while those who prefer in-kind benefits prefer in-kind benefits.

### b. Results

Table 3 shows the results of the model based on Eq (1) where the dependent variable is respondent choosing in-kind benefit worth NRs. 1,000 over NRs. 1,000 cash (In a separate model specification, we ran a model without district fixed effect. Although having similar results, the R-squared value as a measure of goodness of fit suggests that the use of area fixed effect performs better. Thus, we report the model with area fixed effect or simply controlling for the districts dummies). We highlight only significant results here. The individual with higher off-farm income by one hundred thousand NRs, the respondents are less likely to choose in-kind benefits by 0.2%. In terms of magnitude, the result is not significant, but it holds a significant value. High-income households’ preference for cash can be explained by the fact that they could have purchased enough of the goods that is required in a household—which makes those households choose cash instead. Surprisingly, those who don’t have NRs. 25 thousand savings for emergency, they are more likely to choose in-kind transfers by around 10%. These two estimates suggest that lower chance of having cash in the households make the individuals choose in-kind benefits instead of cash benefits. This result could be due to the fact that low-income households are looking for in-kind benefits that could be useful for them to meet their daily ends. These results do not support the fact that low income households could use cash to spend in risky consumption (like alcohol and cigarettes) as is shown in some of the previous studies [18, 19].

**Table 3:** Determinants of choosing in-kind benefit over cash of equal value in a study among Nepalese farmers in 2023.

	Estimates	Robust SE
Female	0.032	0.032
*Higher* caste	-0.042	0.033
High school or more	0.012	0.034
Off farm income	-0.002***	0.0001
Lack NRs. 25,000 saving	0.097***	0.036
Low quality goods and services	-0.054	0.042
Food insecurity	-0.033	0.038
Market distance	0.018*	0.009
Have received cash benefit	-0.106**	0.042
Have received in kind benefit	0.087***	0.033
Areas (Ref Chitwan)		
Kathmandu valley	0.0001	0.054
Kapilvastu	0.021	0.058
Palpa	-0.062	0.045
Rupandehi	-0.088	0.054
Constant	0.598***	0.054
Observations	954	
R-squared	0.039	

***, **, and * indicate level of significance for less than 1%, 5%, and 10%, respectively. The result is based on linear probability model (LPM) because of its flexibility and convenient interpretation of the estimation.

The results show that those who live farther from the market would prefer in-kind transfers. Living 10 minutes away from the market would decrease the choice of in-kind benefit over cash benefit by 2%. This relationship is consistent with Ghatak et al. [14]. This result could be because the farther the market the more transaction cost (time cost and transportation cost) is required to procure the goods. Thus, households with less market access could find beneficial to choose in-kind benefits. Quite expectedly, receiving cash benefit and in-kind benefits previously would make them choose cash and in-kind benefits, respectively. Past experience of receiving cash benefit (or in-kind benefit) would decrease (or increase) the likelihood of choosing in-kind benefit by around 10%. The constant term is an intercept term which can be interpreted as a share of respondents who choose in-kind benefit if all variables are fixed zero.

Further, in the second part of the experiment, we surveyed only those who responded that they would choose NRs. 1,000 cash instead of an in-kind benefit worth of NRs. 1,000. Table 4 shows the results by the value of the in-kind benefits and the type of the in-kind benefit proposed. This result based on descriptive analysis.

**Table 4:** Preference of rice and fertilizer of different values vs NRs. 1000 cash in a study conducted on the determinants of choosing in-kind benefits over cash among Nepalese farmers in 2023.

Value of in-kind (NRs.)	Respondents choosing in-kind (%)		
	Rice (n = 162)	Fertilizer (n = 158)	Total (N = 320)
1150	38	53	46
1300	43	58	51
1450	45	50	47
Aggregate	42	54	48

Note: This experiment is based on only those who choose cash instead of in-kind benefit in the first stage of the experiment.

Even after increasing the valuation of the in-kind benefit to make it equivalent up to NRs. 1,450 (ranging from NRs 1150 to NRs 1450), only 48% preferred in-kind transfers. Surprisingly, only 42% of the households prefer rice over 1,000 NRs. cash, even when the value of the offered rice was more than NRs. 1,000. 58% of the respondents would forego higher valuation in-kind benefit for cash benefit. Likewise, when fertilizer is proposed instead of rice, 54% respondents would choose fertilizer worth more than NRs. 1,000. For fertilizer, almost half of the respondents would forgo fertilizer worth more than NRs. 1,000 for NRs. 1,000 cash. During the survey period, there was a severe shortage of fertilizers in Nepal, which made a larger share of respondents choose in-kind benefit when the proposed in-kind benefit is fertilizer compared to those assigned randomly to choose between cash and rice as an in-kind benefit. Although the severity of the fertilizer shortage during the survey time is worth reporting given the chronic problem in Nepalese fertilizer supply chain [20], this result should not be viewed as a surprise.

Back of the envelope calculation shows that by choosing cash over higher value in-kind benefit 52% of the respondents (N = 320) forgo on average NRs. 300. In other words, instead choosing NRs. 1,300 in kind, they choose NRs. 1,000 in cash—losing almost a quarter (25%) of the benefit. This result is comparable with Skoufias et al. [3].

## 6. Conclusion

When providing assistance to resource-poor individuals through in-kind or cash transfers, it is crucial to understand the recipients’ preferences. While theoretical considerations suggest that individuals are better off choosing cash over in-kind transfers of equivalent value, empirical evidence reveals that several factors influence recipients’ decisions. In this study, we conducted an experiment among farmers in Nepal, offering them a choice between NRs. 1,000 in cash and in-kind transfers of equal or more value. Surprisingly, approximately two-thirds of the respondents opted for in-kind benefits of equal value.

Our findings indicate that households with off-farm income and previous experience receiving cash benefits are less likely to choose in-kind transfers. Conversely, households with limited savings, living farther from the market, and prior exposure to in-kind benefits are more inclined to choose in-kind benefits. In a second step of the experiment, we hypothetically explored whether those who initially chose cash would switch to a higher-valued in-kind benefit. Only 48% of respondents opted for the in-kind benefit when its value exceeded NRs. 1,000. Remarkably, 52% of participants were willing to forgo a significant portion of the in-kind benefit in favor of a lower-valued cash transfer. The type of the in-kind benefit would also affect whether the respondents would choose in-kind benefit over cash, even if the market value of the in-kind commodity is larger than the proposed cash option.

This study provides an insight why some recipients might not choose cash over in-kind benefits of same value, and that has some policy implications. People in remote areas might increase their welfare if they are provided with in-kind benefits while it could not be the case for people in more accessible areas. Further, people who are more likely to have cash could benefit by having cash as a benefit as they could utilize the cash to meet their needs. For instance, they could already have managed fertilizer or seeds upfront, however, extra cash could be spent on non-agricultural sectors. As our second experiment suggested that more people would choose fertilizer than rice if the value of these in-kind benefits are larger than the proposed cash benefit, this result could be of policy implication as ag-inputs (which was shortage at the survey time) could be of benefit of choice to the people.

Despite its potential contribution to the development economics literature, this study has some limitations. Despite our effort to control it, there could have been a few instances of spillover of the experimental setup for some participants affecting the estimates. However, we believe such an effect to be minimum. Further, this study is not causal study, making the relationship between variables and choice of in-kind benefit a correlation rather than a causation. Thus, the interpretation of the estimates should be made with caution. Further, this study represents the farming households in Central and Western Nepal. While the study makes the sampling representative of the farming population of Nepal, the results may not be generalized in the context of other developing economy. Future studies should delve into this topic. It is important to note that this study solely focuses on recipient preferences and does not explore how the benefits are utilized or the welfare impact within recipient households. A long-term study with higher-value support is necessary to address these aspects comprehensively. Additionally, future studies should factor in the distribution costs associated with cash and in-kind transfers.

In summary, our findings underscore the diverse needs of potential recipients, highlighting the inadequacy of a one-size-fits-all approach. A tailored approach in implementing assistance programs should be applied that could be based on income level and accessibility to the market. Therefore, a thorough understanding of farmers’ needs is imperative before implementing programs.
